# Supplementing with Non-Glycoside Hydrolase Proteins Enhances Enzymatic Deconstruction of Plant Biomass

**DOI:** 10.1371/journal.pone.0043828

**Published:** 2012-08-27

**Authors:** Xiaoyun Su, Jing Zhang, Roderick I. Mackie, Isaac K. O. Cann

**Affiliations:** 1 Energy Biosciences Institute, University of Illinois, Urbana, Illinois, United States of America; 2 Institute for Genomic Biology, University of Illinois, Urbana, Illinois, United States of America; 3 Department of Animal Sciences, University of Illinois, Urbana, Illinois, United States of America; 4 Department of Microbiology, University of Illinois, Urbana, Illinois, United States of America; University of Waikato, New Zealand

## Abstract

The glycoside hydrolases (GH) of *Caldicellulosiruptor bescii* are thermophilic enzymes, and therefore they can hydrolyze plant cell wall polysaccharides at high temperatures. Analyses of two *C. bescii* glycoside hydrolases, CbCelA-TM1 and CbXyn10A with cellulase and endoxylanase activity, respectively, demonstrated that each enzyme is highly thermostable under static incubation at 70°C. Both enzymes, however, rapidly lost their enzymatic activities when incubated at 70°C with end-over-end shaking. Since crowding conditions, even at low protein concentrations, seem to influence enzymatic properties, three non-glycoside hydrolase proteins were tested for their capacity to stabilize the thermophilic proteins at high temperatures. The three proteins investigated were a small heat shock protein CbHsp18 from *C. bescii*, a histone MkHistone1 from *Methanopyrus kandleri*, and bovine RNase A, from a commercial source. Fascinatingly, each of these proteins increased the thermostability of the glycoside hydrolases at 70°C during end-over-end shaking incubation, and this property translated into increases in hydrolysis of several substrates including the bioenergy feedstock Miscanthus. Furthermore, MkHistone1 and RNase A also altered the initial products released from the cello-oligosaccharide cellopentaose during hydrolysis with the cellodextrinase CbCdx1A, which further demonstrated the capacity of the three non-GH proteins to influence hydrolysis of substrates by the thermophilic glycoside hydrolases. The non-GH proteins used in the present report were small proteins derived from each of the three lineages of life, and therefore expand the space from which different polypeptides can be tested for their influence on plant cell wall hydrolysis, a critical step in the emerging biofuel industry.

## Introduction

Plant cell wall polysaccharides, a promising resource for producing renewable biofuels, are the most abundant biomass on earth [1]. The plant cell wall is recalcitrant, and requires an array of glycoside hydrolases to deconstruct it into simple sugars for fermentation to biofuels [2], [3]. In nature, extracellular and intracellular microbial glycoside hydrolases function synergistically to release sugars from biomass [4], [5]. The secreted enzymes break down the polysaccharides into oligosaccharides with different degrees of polymerization. The oligosaccharides are then transported into the cell by transporters embedded in the cell wall. Next, intracellular glycoside hydrolases degrade the oligosaccharides into simple sugars, which are readily utilized by the organism.

The intracellular environment is crowded with macromolecules, with a concentration as high as 50–400 mg/ml [6]. Accordingly, the biochemical properties of intracellular proteins may differ from those obtained in routine laboratory assays. A “molecular crowding” environment has been demonstrated to affect a number of biochemical characteristics of intracellular proteins, such as enzymatic activity [7], [8] and DNA-binding [9]. Even at low concentrations, such as 0.1% (or 1 mg/ml), of crowding agents, the interaction between proteins and crowding agents may still be significant [7]. It is also known that the behavior of extracellular proteins can be affected. For example, formation of amyloid fiber by a human apolipoprotein is accelerated by macromolecular crowding [10].

Currently the glycoside hydrolases used in biofuel are mainly from the mesophilic filamentous fungus *Trichoderma reesei* [11], [12]). The cost for such enzymes has been substantially lowered in the last few years. In spite of this achievement, there is still a vast interest in developing thermophilic glycoside hydrolases as alternative enzymes. However, the production levels of heterologous thermophilic enzymes may, at least in some cases, be low. For example, the expression of a *Dictyoglomus thermophilum* xylanase failed in *T. reesei*. After codon optimization, the xylanase was expressed and a halo could be observed around the transformant colonies in a plate assay [13]. The thermophilic glycoside hydrolases of *Caldicellulosiruptor bescii* are excellent candidates for application in the biofuel industry [14], [15]. A bioinformatics analysis revealed a reservoir of up to 88 putative CAZy (Carbohydrate-Active enZYmes, http://www.cazy.org) genes in the *C. bescii* genome [16]. Among the genes are those encoding secreted as well as those coding for intracellular enzymes. In our hands, the yield of a robust *C. bescii* GH51 α-arabinofuranosidase in *E. coli* is as low as ∼0.8 mg/L in batch culture (Cann et al., unpublished data). Therefore, the cost of thermophilic glycoside hydrolases could impede its application in biofuel.

In this work, we investigated how the presence or addition of proteins lacking glycoside hydrolase activity (non-GH) will impact the glycoside hydrolases of the thermophilic bacterium *C. bescii* during hydrolysis of plant cell wall polysaccharides. The non-GH proteins used in this paper are small proteins, which may ease the process of heterologous production. In the present study, we added three non-GH proteins [a small heat shock protein CbHsp18 from *C. bescii*, a histone (MkHistone1) from a hyperthermophilic archaeon *Methanopyrus kandler*i, and bovine RNase A] to three thermophilic glycoside hydrolases and investigated their individual effects on hydrolysis of either plant cell wall polysaccharides or a model cello-oligosaccharide. The three proteins were chosen from the three lineages of life, and thereby increasing the range of proteins that could be selected. Moreover, CbHsp18 and MkHistone1 are from thermophilic microorganisms, and it was hypothesized that they would be thermostable. Although RNase A is from a mesophilic source, it is well-known for its thermostability.

Motivated by the fact that low concentrations (such as 1 mg/ml [7]) of crowding proteins can still significantly affect the behavior of a glucosidase, we sought to find out if adding low amounts of non-GH proteins will have beneficial effects on the hydrolysis of plant cell wall polysaccharides by *C. bescii* glycoside hydrolases. The highest concentrations of CbHsp18, MkHistone1, and RNase A were 1.26, 1.22, and 0.88 mg/ml (corresponding to 64 µM) for analyses of the effect of the non-GH proteins on a binary glycoside hydrolase-mixture. These concentrations approximate the 1 mg/ml shown to stimulate the glucosidase mentioned above. Because low concentrations of proteins are favored for industrial application, we further extended the use of non-GH proteins to two even lower concentrations.

While all three proteins can be regarded as crowding proteins, CbHsp18 was unique because it belongs to a family of small heat shock proteins that are known to function as molecular chaperones, and therefore minimize protein denaturation and/or aggregation by unfavorable chemical reagents or physical factors [17], [18]. Since a thorough mixing of enzymes with substrates is needed for maximal deconstruction of insoluble plant biomass [19], [20], an end-over-end shaking incubation approach was used to investigate hydrolysis of the model feedstock Miscanthus and crystalline cellulose Avicel. Furthermore, we investigated the effects of each of the three proteins on the thermostability of the thermophilic plant cell wall degrading enzymes from *C. bescii*. The studies reported here should provide insights into enhancing the capacity to harness enzymes for release of fermentable sugars from plant biomass for subsequent fermentation to biofuels.

## Results

### Cloning, Expression, and Purification of CbCelA-TM1, CbCdx1A, CbXyn10A, CbHsp18, and MkHistone1

**Figure 1:**
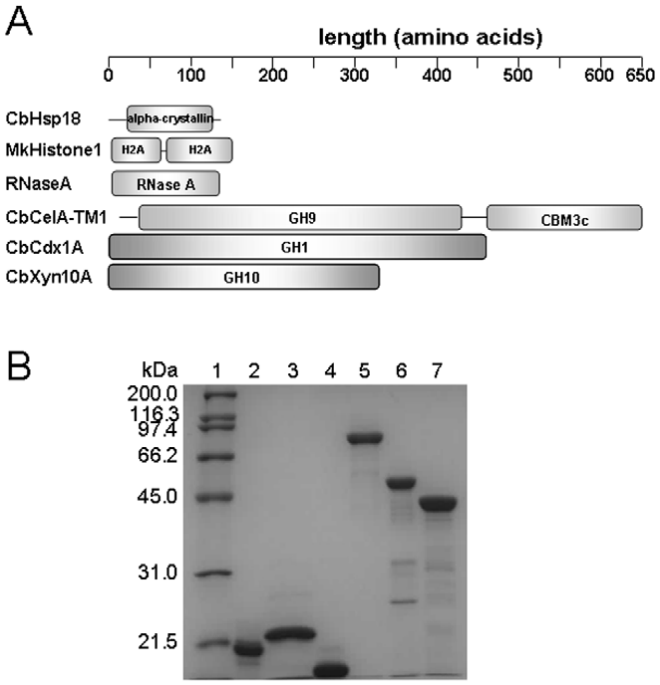
Schematic domain structures (A) and SDS-PAGE analysis (B) of the non-GH proteins and glycoside hydrolases. A: A schematic representation of the polypeptides of CbHsp18, MkHistone1, and the three glycoside hydrolases of *C. bescii* used in this study. GH9: Glycoside hydrolase family 9 domain; CBM3c: Carbohydrate binding module family 3 type C. B: SDS-PAGE analysis of purified recombinant non-GH proteins and the three glycoside hydrolases of *C. bescii*. Lane 1: molecular mass markers; lane 2: CbHsp18; lane 3: MkHistone1; lane 4: RNase A; lane 5: CbCelA-TM1; lane 6: CbCdx1A; lane 7: CbXyn10A. Two micrograms of each protein were resolved by 12% SDS-PAGE. RNase A was a commercial product (Roche).

Analysis of the genome of the hyperthermophilic bacterium *C. bescii* yielded genes encoding five putative endoglucanases, one putative cellodextrinase, and six putative xylanases. The five endoglucanases are predicted to be secreted proteins since each translated product contained an N-terminal signal peptide. In contrast, the cellodextrinase CbCdx1A is predicted to be intracellularly located. Indeed, it has been reported that the polypeptide from which CbCelA-TM1 is derived, i.e., CbCel9A/Cel48A (or CelA) is the most abundant glycoside hydrolase in Avicel-induced *C. bescii* culture supernatant [21]. The full length polypeptide of CbCel9A/Cel48A failed to express in *E. coli* cells. Therefore, we cloned and expressed a truncated mutant composed of the N-terminal GH9 catalytic module with a carbohydrate binding module 3c (Fig. 1A). This truncated polypeptide with cellulase activity was designated CbCel9A/Cel48A-TM1. For ease of understanding, we will refer to this protein as CbCelA-TM1. The cellodextrinase CbCdx1A, with a GH1 catalytic module, an intracellularly located xylan-degrading enzyme CbXyn10A, with a GH10 catalytic module, a small heat shock protein from *C. bescii* with an alpha-crystalline domain, and a thermostable histone protein, with two histone 2A (H2A) modules, from *Methanopyrus kandleri* (Fig. 1A) were also expressed in *E. coli* and purified. The third non-GH protein, RNase A, was purchased from Roche Applied Science (Indianapolis, IN). The purified recombinant proteins of CbCelA-TM1, CbCdx1A, CbXyn10A, and CbHsp18 were resolved on SDS-PAGE, and each protein exhibited a molecular mass that corresponded closely to the calculated values based on the polypeptide sequence (Fig. 1B). The recombinant MkHistone1 showed a slightly larger molecular mass of ∼22 kDa than calculated 19.2 kDa (Fig. 1B). As MkHistone1 is from a hyperthermophile which can grow at a temperature up to 110°C [22], it was hypothesized that part of its secondary structure might have failed to fully denature in the SDS-PAGE analysis and hence the slightly larger molecular mass on the gel.

### Non-GH Proteins Increase Stability of CbCelA-TM1 at High Temperature and Increase Hydrolysis of Cellulose

**Figure 2:**
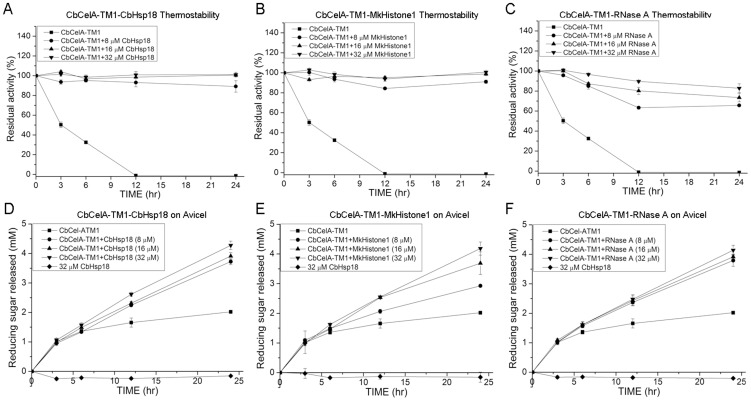
Thermostability of CbCelA-TM1 in presence of CbHsp18 (A), MkHistone1 (B), and RNase A (C) and hydrolysis of Avicel with CbCelA-TM1 in presence of CbHsp18 (D), MkHistone1 (E), and RNase A (F). For the thermostability assay, 1 µM of CbCelA-TM1 was incubated with 8 µM, 16 µM, or 32 µM of CbHsp18, MkHistone1, or RNase A in a pH 6.0 citrate buffer. The mixtures were shaken end-over-end at 70°C for 24 hr. As a control, 1 µM of CbCelA-TM1 in the same buffer was incubated without shaking at 4°C. For hydrolysis of Avicel, 1 µM of CbCelA-TM1 was incubated with 20 mg/ml Avicel in the absence or presence of 8 µM, 16 µM, or 32 µM of CbHsp18, MkHistone1, or RNase A in a pH 6.0 citrate buffer. The reaction mixtures were shaken end-over-end at 70°C for 24 hr.

As *C. bescii* is a bacterium with an optimal growth temperature of 75°C [23], its glycoside hydrolases may be regarded as either thermophilic or hyperthermophilic proteins. CbCelA-TM1 incubated without agitation was thermostable at 70°C as it retained 74.5±3.9% of its initial enzymatic activity after 24 hr (data not shown). The enzyme, however, lost its activity rapidly when the samples were shaken end-over-end at 70°C (Fig. 2A, B, and C). After 3 hr and 6 hr incubation, the residual activities of CbCelA-TM1 were 50.2±2.8% and 32.5±1.6%, respectively. After 12 hr, CbCelA-TM1 completely lost its activity under this condition. As expected, the small heat shock protein CbHsp18 dramatically increased the stability of CbCelA-TM1 under the end-over-end shaking conditions. Adding 8 µM, 16 µM, and 32 µM of CbHsp18 increased the residual activity to 89.1±5.8%, 100.7±1.4%, and 101.1±2.3%, respectively after 24 hr of incubation at 70°C (Fig. 2A). Interestingly, MkHistone1 and RNase A could also increase the stability of CbCelA-TM1. Adding 8 µM, 16 µM, and 32 µM of MkHistone1 increased the residual activity of CbCelA-TM1 to 91.1±1.7%, 98.8±1.5%, and 100.7±1.2%, respectively (Fig. 2B), and adding 8 µM, 16 µM, and 32 µM of RNase A increased the residual activity of CbCelA-TM1 to 65.7±1.1%, 73.5±4.2%, and 82.8±4.4%, respectively, after 24 hr incubation at 70°C (Fig. 2C).

In a time course experiment, where hydrolysis of the model crystalline cellulose Avicel was analyzed, CbCelA-TM1 released 1.0±0.1 mM of reducing ends after 3 hr, and after 24 hr only about 2.0±0.1 mM reducing ends could be detected, suggesting very slow release of end products after 3 hr (Fig. 2D, E, and F). None of the three added proteins could increase the release of reducing sugar in the first 3 hr, indicating that the initial activities for these reactions were not affected. Adding CbHsp18, MkHistone1 or RNase A to CbCelA-TM1, however, led to increased release of reducing ends from 3 hr to 24 hr. The highest concentration of all three non-GH proteins had the largest enhancing effect, with 4.3±0.1 mM reducing sugars released by CbCelA-TM1 in presence of 32 µM of CbHsp18 (Fig. 2D), 4.2±0.2 mM in presence of 32 µM of MkHistone1 (Fig. 2E), and 4.1±0.2 mM in presence of 32 µM of RNase A (Fig. 2F). Significant difference appears between 16 µM and 32 µM CbHsp18 (*p*<0.01) and between 8 µM and 16 µM MkHistone1 (*p*<0.01), but not between 8 µM and 16 µM of CbHsp18, 16 µM and 32 µM of MkHistone1, and not among different concentrations of RNase A.

The enhanced hydrolysis of Avicel in the presence of each of the three proteins was further confirmed by HPLC analysis. The end products of all Avicel hydrolysis after 24 hr incubation with CbCelA-TM1 with or without the non-GH proteins were glucose and cellobiose (Table 1). In presence of all three concentrations of CbHsp18, MkHistone1, or RNase A, releases of both glucose and cellobiose were enhanced. Generally, higher concentrations of glucose and cellobiose were released with increasing concentrations of the non-GH proteins (Table 1), although this trend was not so obvious for the hydrolysis in presence of 16 µM and 32 µM of MkHistone1 and RNase A. The hydrolysis pattern was not affected since the cellobiose/glucose ratios were similar for reactions with and without the non-GH proteins (Table 1). It was concluded that adding each of the three proteins increased hydrolysis of crystalline cellulose by stabilizing CbCelA-TM1 at the high temperature.

**Table 1:** End products from hydrolysis of Avicel by CbCelA-TM1 in the presence or absence of non-GH proteins^a^.

Glycoside hydrolase	Non-GH Protein	Protein concentration (µM)	Glucose (mM)	Cellobiose (mM)	Glucose/cellobiose ratio
CbCelA-TM1	n/a	n/a	1.3±0.0	1.5±0.1	1.2
	CbHsp18	8	2.1±0.0	2.5±0.0	1.2
		16	2.2±0.0	2.8±0.0	1.3
		32	2.3±0.1	3.0±0.2	1.3
	MkHistone1	8	1.7±0.1	2.1±0.1	1.2
		16	2.3±0.1	2.8±0.1	1.2
		32	2.3±0.0	2.7±0.0	1.2
	RNase A	8	2.0±0.1	2.4±0.0	1.2
		16	2.2±0.2	2.7±0.2	1.2
		32	2.3±0.1	2.8±0.0	1.2

a The hydrolysis was carried out at 70°C for 24 hr under an end-over-end shaking condition. The hydrolysis products after 24 hr were analyzed by HPLC. GH: glycoside hydrolase.

### MkHistone1 and RNase A Change the Hydrolysis Pattern of Cellopentaose by CbCdx1A

**Figure 3:**
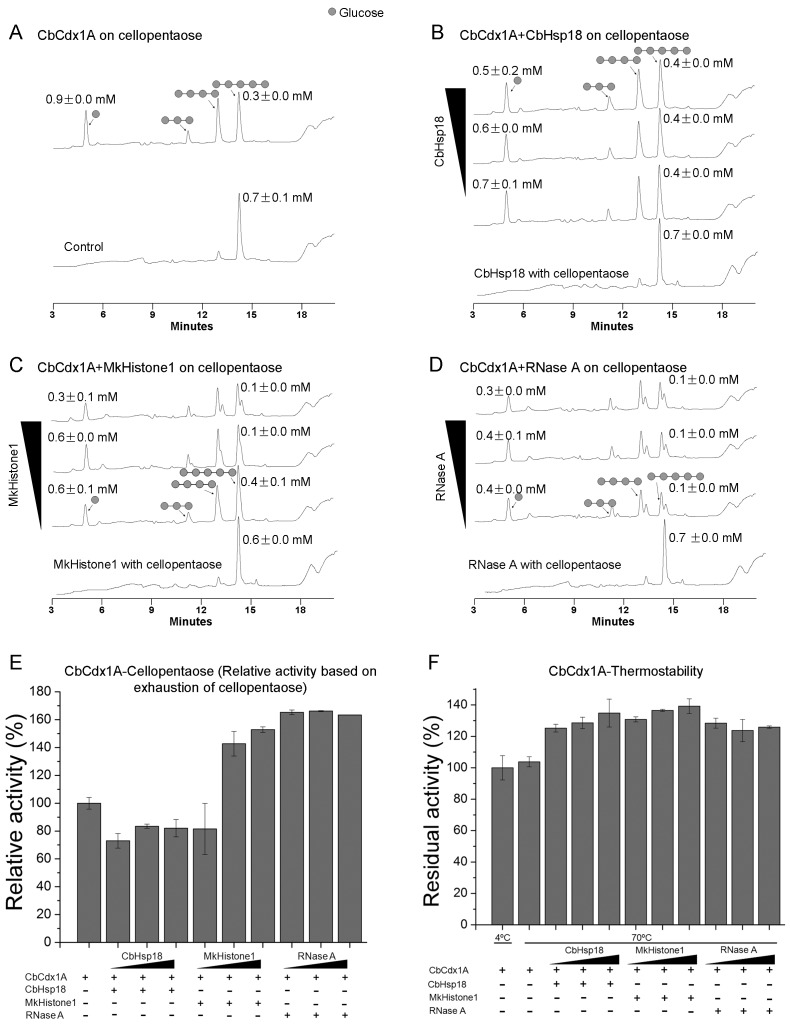
Hydrolysis of cellopentaose by CbCdx1A in the absence or presence of non-GH proteins. A: Hydrolysis of cellopentaose byCbCdx1A in absence of the non-GH proteins. Control: cellopentaose incubated alone; CbCdx1A: cellopentaose incubated with 1 µM of CbCdx1A. B–D: Hydrolysis of cellopentaose in the presence of 8 µM, 16 µM, and 32 µM of CbHsp1 (B), MkHistone1 (C), or RNase A (D). For these reactions, cellopentaose (0.68 mM) was incubated with 1 µM of CbCdx1A in the absence or presence of the non-GH proteins at 70°C for 9 min. The cellopentaose was also incubated individually with each of the three non-GH proteins (32 µM). The samples were then immediately diluted 100 times with HPLC buffer (100 mM NaOH) to inactivate the enzyme and subjected to HPLC analysis. E: The activity of CbCdx1A in absence of non-GH proteins was set as 100%, and the relative activities from the other reactions were calculated by dividing their corresponding activities with this reference activity. F: Thermostability of CbCdx1A at 70°C. One micromolar of CbCdx1A was shaken end-over-end at 70°C in the absence or presence of 8 µM, 16 µM, or 32 µM of CbHsp1, MkHistone1, or RNase A in a volume of 500 µl; at the same time, 1 µM of CbCdx1A was kept incubated without shaking at 4°C as a control. After 24 hr, the residual activities were measured for all samples using *p*-NP-β-D-glucopyranoside as the substrate. The activity of the sample at 4°C was set as 100%, and the residual activities for the other samples were calculated by dividing their activities by that of the sample incubated at 4°C.

CbCelA is a secreted cellulase and is expected to act on cellulose outside of the cell. In contrast, CbCdx1A is an intracellular cellodextrinase that is responsible for cleaving cello-oligosaccharides into glucose. Unlike CbCelA-TM1, CbCdx1A is highly thermostable even under end-over-end shaking conditions, as it exhibits 103.8±3.2% residual activity after incubation at 70°C for 24 hr (Fig. 3F). It was surprising that adding the three non-GH proteins under investigation appeared to further increase the residual activities of CbCdx1A. Thus in the presence of 8 µM, 16 µM, and 32 µM CbHsp18, the residual activities recorded were 125.3±2.5%, 128.6±3.7%, and 134.8±8.9%, respectively. The residual activities in the presence of 8 µM, 16 µM, and 32 µM of MkHistone1 were 130.8±1.6%, 136.6±0.6%, and 139.3±4.7%, respectively, and the values in presence of 8 µM, 16 µM, and 32 µM RNase A were 128.5±3.2%, 123.8±7.0%, and 125.9±0.8%, respectively (Fig. 3F).

In the initial hydrolysis (9 min) of cellopentaose by CbCdx1A, cellopentaose was hydrolyzed mainly into cellotetraose and glucose, with minor cellotriose (Fig. 3A). Incubation of cellopentaose with 32 µM of CbHsp18, MkHistone1, or RNase A did not result in hydrolysis of cellopentaose into smaller cello-oligosaccharides (Fig. 3B, C, and D). The hydrolysis pattern did not change with any of the concentrations of CbHsp18 (8 µM, 16 µM, or 32 µM) (Fig. 3B) and also MkHistone1 at 8 µM (Fig. 3C). However, a peak with a retention time of 13.31 min lagging after cellotetraose and another peak with a retention time of 14.49 min after cellopentaose clearly appeared when higher concentrations (16 µM and 32 µM) of MkHistone1 were tested (Fig. 3C). These peaks formed a shoulder with those of cellotetraose and cellopentaose in the HPLC analysis, and they appeared in the reactions for all three concentrations of RNase A (Fig. 3D). The nature of these new peaks was not further investigated in this study.

It was further found that the hydrolysis of cellopentaose decreased for all CbCdx1A-CbHsp18 reactions (72.9±5.2%, 83.5±1.6%, and 82.1±6.2% for 8 µM, 16 µM, and 32 µM of CbHsp18, respectively, Fig. 3E) compared with that of CbCdx1A alone (set as 100%). Except for the lowest concentration (8 µM), the hydrolysis of cellopentaose increased with addition of MkHistone1 and all three concentrations of RNase A (Fig. 3E). No hydrolyzing activity was observed for any of the three non-GH proteins. Note, however, that the increased hydrolysis of cellopentaose did not result in higher accumulation of glucose (Fig. 3A–D).

### Hydrolysis of Avicel by a Binary Cellulase Mixture Containing CbCelA-TM1 and CbCdx1A

**Figure 4:**
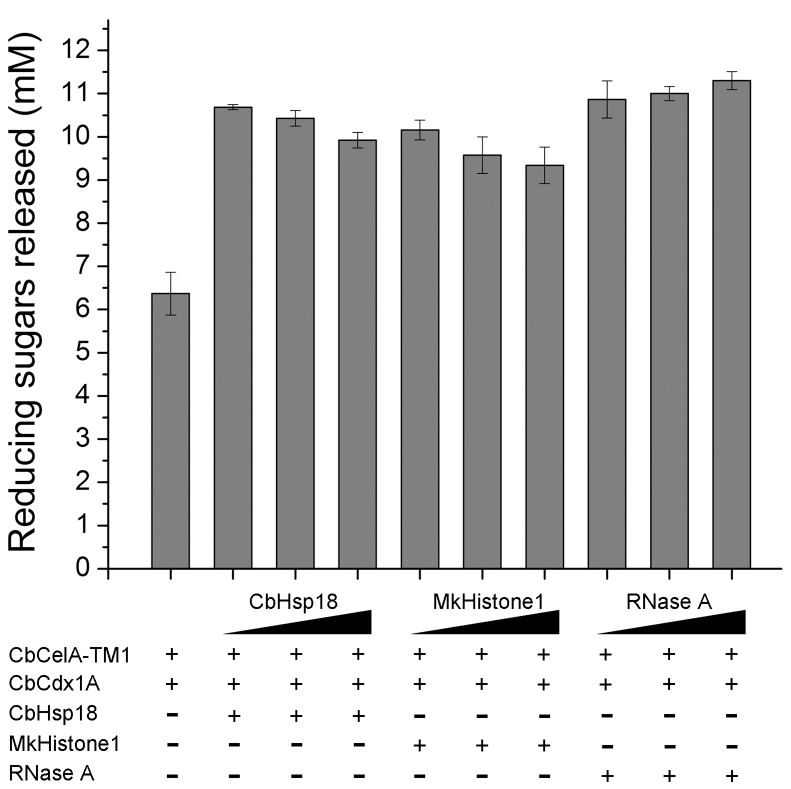
Hydrolysis of Avicel by a binary mixture of CbCelA-TM1 and CbCdx1A. Avicel (20 mg/ml) was hydrolyzed with 1 µM each of CbCelA-TM1 and CbCdx1A in the absence or presence of 16 µM, 32 µM, and 64 µM of CbHsp18, MkHistone1, or RNase A in a pH 6.0 citrate buffer at a total volume of 500 µl. The reactions were carried out by shaking the mixtures end-over-end at 70°C for 24 hr. The reducing ends released in all samples were determined by the PAHBAH method.

The endoglucanase CbCelA-TM1 and cellodextrinase CbCdx1A can synergistically act in hydrolyzing cellulose. The binary cellulase mixture containing CbCelA-TM1 and CbCdx1A released 6.4±0.5 mM of reducing sugars after 24 hr (Fig. 4), much more than the amounts released by CbCelA-TM1 alone (2.0±0.1 mM, Fig. 2D, E, and F) or CbCdxlA alone (too low to accurately measure, data not shown). Addition of CbHsp18, MkHistone1, and RNase A all further increased the hydrolysis of Avicel. In this case, there was no increasing trend in release of reducing sugars with increasing concentrations of the non-GH proteins, which was in contrast to the observation made for Avicel hydrolysis by CbCelA-TM1 alone (Fig. 4). The enhancing effect of the three proteins on end product release by the glycoside hydrolases was further confirmed by HPLC analysis. Glucose was the only end product in all the reactions (Table 2), indicating that CbCdx1A converted all the cellobiose produced by CbCelA-TM1 to glucose. There was no statistical significance in the differences in the amounts of glucose released in the reactions at the three concentrations of CbHsp18. This observation also applied to the reaction with MkHistone1 and RNase A (Table 2). Despite the lack of statistical significance, the data suggested that under the conditions tested, RNase A showed the best enhancing effect (Table 2, Figure 4).

**Table 2:** End products of hydrolysis of Avicel by CbCelA-TM1/CbCdx1A in the presence or absence of non-GH proteins^a^.

Glycoside hydrolases	Non-GH protein	Protein concentration (µM)	Glucose (mM)
CbCelA-TM1+CbCdx1A	n/a	n/a	7.4±0.5
	CbHsp18	16	10.9±0.7
		32	11.1±0.5
		64	10.8±0.5
	MkHistone1	16	10.8±0.0
		32	10.8±0.3
		64	11.0±0.0
	RNase A	16	12.1±0.1
		32	12.1±0.1
		64	12.1±0.3

a The hydrolysis was carried out at 70°C for 24 hr under an end-over-end shaking condition. The hydrolysis products after 24 hr were analyzed by HPLC. GH: glycoside hydrolase.

### Hydrolysis of a Bioenergy Feedstock Miscanthus by CbXyn10A is Enhanced by CbHsp18, MkHistone1, and RNase A

**Figure 5:**
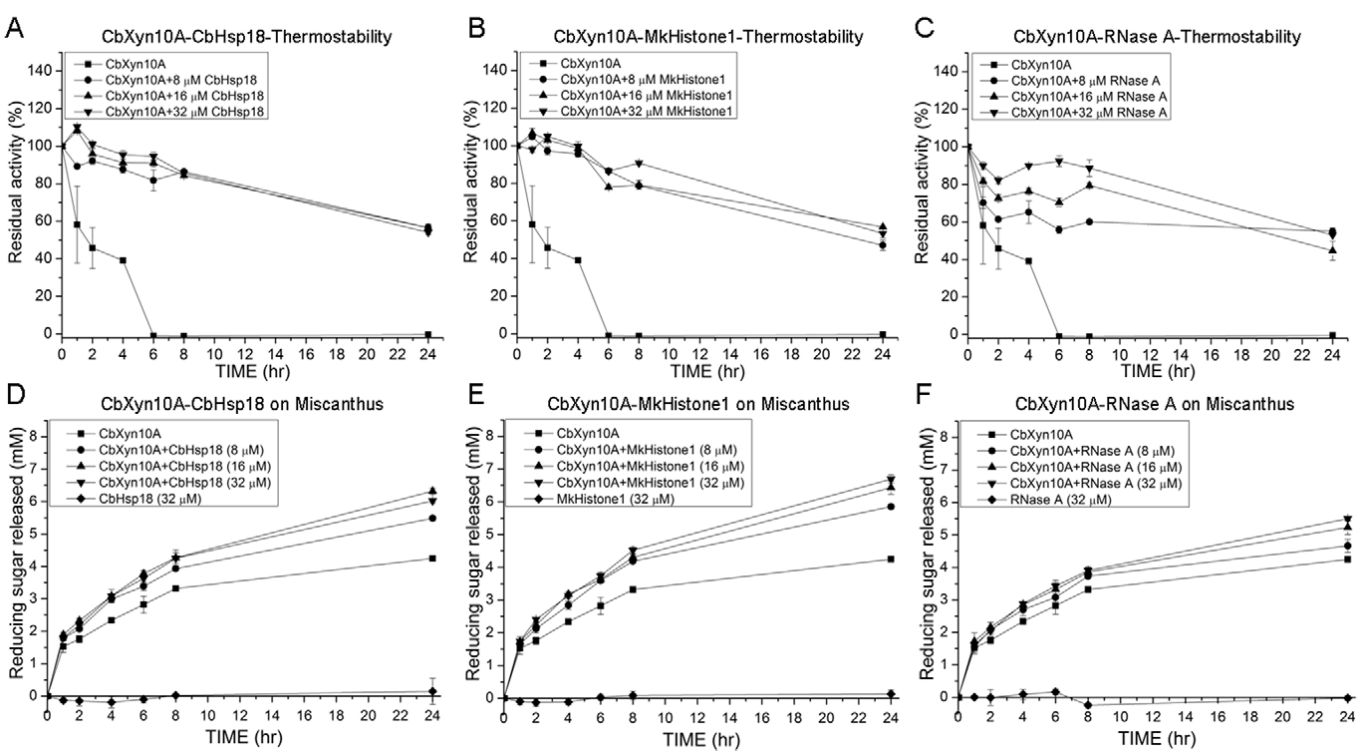
Thermostability of CbXyn10A (A, B, and C) and hydrolysis of Miscanthus by CbXyn10A (D, E, and F). A–C: Thermostability of CbXyn10A in the presence of CbHsp18 (A), MkHistone1 (B), and RNase A (C). One micromolar of CbXyn10A was incubated with 8 µM, 16 µM, or 32 µM of CbHsp18, MkHistone1, or RNase A in a pH 6.0 citrate buffer in a total volume of 500 µl. The mixtures were shaken end-over-end at 70°C for 24 hr. D–E: 1 µM of CbXyn10A was incubated with 20 mg/ml Miscanthus in the absence or presence of 8 µM, 16 µM, and 32 µM each of CbHsp18 (D), MkHistone1 (E), and RNase A (F) in a pH 6.0 citrate buffer in a total volume of 500 µl. The reaction mixtures were rotated end-over-end at 70°C for 24 hr.

Bioenergy feedstocks such as Miscanthus and switchgrass contain hemicellulose in addition to cellulose. Endoxylanases constitute a critical group of enzymes needed for hydrolysis of hemicelluloses, such as xylan, since they cleave the β-1,4 glycosidic linkages of the backbone of the polysaccharide. The hydrolytic products are shorter chain sugars that can be further hydrolyzed to simple sugars such as xylose and arabinose. We, therefore, analyzed the effects of the three non-GH proteins on the thermostability and xylan hydrolysis by a *C. bescii* endoxylanase CbXyn10A at 70°C. CbXyn10A was less thermostable than CbCelA-TM1 and CbCdx1A; however, CbXyn10A still maintained 47.5±1.9% residual activity when incubated at 70°C for 24 hr without shaking (data not shown). CbXyn10A was much more unstable under an end-over-end shaking condition at 70°C. Under this condition, the endoxylanase rapidly lost its activity, retaining 58.2±20.5%, 45.7±10.9%, and 39.0±0.3% residual activity after 1 hr, 2 hr, and 4 hr incubation, respectively (Fig. 5A, B, and C). The endoxylanase completely lost its activity after 6 hr (Fig. 5A, B, and C). Adding any of the three small proteins (CbHsp18, MkHistone1 or RNase A) provided some thermostability to CbXyn10A under end-over-end shaking. After 24 hr, the residual activities of CbXyn10A in presence of 8 µM, 16 µM, and 32 µM CbHsp18 were 56.5±1.7%, 56.5±1.9%, and 54.0±1.2%, respectively (Fig. 5A). When MkHistone1 was added at 8 µM, 16 µM, and 32 µM, the residual activities were 47.0±2.8%, 56.8±0.3%, and 53.3±2.3%, respectively, after a 24 hr incubation (Fig. 5B), and similar observations were made for addition of 8 µM, 16 µM, and 32 µM RNase A (Fig. 5C).

The initial hydrolysis of birch wood xylan in a 1-min assay by CbXyn10A was slightly decreased in presence of each of the three non-GH proteins. By setting the activity of CbXyn10A alone as 100%, its activities in presence of 8 µM, 16 µM, and 32 µM CbHsp18, MkHistone1, and RNase A ranged from 79.9±2.8% to 94.7±7.4% (data not shown). In contrast to the short-time assay, the reducing sugars released from a more complex substrate Miscanthus increased by adding CbHsp18 (Fig. 5D), MkHistone1 (Fig. 5E), and RNase A (Fig. 5F) from 2 hr to 24 hr of incubation. At the end of the reaction, the concentration of reducing sugars released by CbXyn10A alone was 4.3±0.1 mM (Fig. 5 D, E, and F), and adding any of the non-GH proteins at the three concentrations (8 µM, 16 µM, and 32 µM) led to increases in the yield of reducing ends ranging from 4.7±0.2 mM (8 µM RNase A, Fig. 5F) to 6.7±0.1 mM (32 µM MkHistone1, Fig. 5E).

The improvement in hydrolysis of Miscanthus was confirmed by HPAEC-PAD analysis of the sugar components in the reaction mixtures. The concentrations of xylose and xylobiose were 0.4±0.2 mM and 1.5±0.0 mM, respectively, for Miscanthus hydrolyzed with CbXyn10A alone. With increasing amounts of any of the non-GH proteins, the concentrations of xylose and xylobiose released by the endoxylanase increased gradually (Table 3). The highest concentrations of xylose were obtained for 32 µM of CbHsp18, MkHistone1, and RNase A, which led to increases of 6.0-, 6.4-, and 5.2-fold, respectively. For xylobiose, the trend was similar to that for xylose, although the maximum increases were only 1.5-, 1.6-, and 1.5-fold, respectively for CbHsp18, MkHistone1, and RNase A. There were relatively small amounts of xylotriose in the reaction mixtures in the absence of the non-GH proteins (0.3±0.0 mM). With CbHsp18, the concentrations of xylotriose were comparable to that of CbXyn10A alone. However, with MkHistone1 and RNase A, the concentrations of xylotriose showed a gradual decrease (Table 3).

**Table 3:** End products of hydrolysis of Miscanthus by CbXyn10A in the presence or absence of non-GH proteins^a^.

Glycoside hydrolase	Non-GH protein	Protein concentration (µM)	Xylose (mM)	Xylobiose (mM)	Xylotriose (mM)
CbXyn10A	n/a	n/a	0.4±0.2	1.5±0.0	0.3±0.0
	CbHsp18	8	1.8±0.1	2.0±0.1	0.3±0.2
		16	2.1±0.1	2.1±0.0	0.2±0.0
		32	2.6±0.0	2.3±0.0	0.4±0.1
	MkHistone1	8	1.9±0.2	2.0±0.2	0.3±0.0
		16	2.2±0.2	2.1±0.1	0.3±0.0
		32	2.8±0.1	2.5±0.0	0.1±0.0
	RNase A	8	1.2±0.1	1.8±0.0	0.2±0.0
		16	1.6±0.0	2.0±0.0	0.2±0.0
		32	2.3±0.2	2.3±0.1	0.1±0.0

a The hydrolysis was carried out at 70°C for 24 hr in an end-over-end shaking condition. The hydrolysis products after 24 hr were analyzed by HPLC. GH: glycoside hydrolase.

## Discussion

In the present study, we show that three non-GH proteins can stabilize thermophilic glycoside hydrolases under a condition (end-over-end shaking) that inactivates their enzymatic activity at a high temperature. There is evidence that mechanical agitation can improve hydrolysis of cellulosic materials [19], [20], [24], [25]. However, the concomitant shearing force may be deleterious to the enzymes used in the hydrolytic reaction [19], [26]. In our case, the glycoside hydrolases CbCelA-TM1 and CbXyn10A from the thermophilic bacterium *C. bescii* were very stable when they were kept static at 70°C. However, the two enzymes rapidly lost their glycosidic linkage-cleaving activities when shaking was introduced into the reaction conditions. It was, therefore, of significance to observe that CbCelA-TM1 and CbXyn10A were very stable in presence of each of the three non-GH proteins even under shaking conditions. This stabilizing effect is likely the underlying reason for the capacity of the non-GH proteinsto enhance hydrolysis of the glycosidic linkages by the glycoside hydrolases. The residual activity of CbCdx1A under shaking condition was not different from that observed under static condition. In both cases the enzyme was very stable (∼100% residual activity). Surprisingly, the residual activities in the presence of each of the non-GH proteins appeared to be higher (Fig. 3F). However, this could be an artifact, i.e. the apparent higher residual activity of CbCdx1A in presence of non-GH proteins may actually come from the measurement process if we take into account that the non-GH proteins changed the hydrolysis pattern and more importantly the velocity of CbCdx1A using cellopentaose as a model substrate in a short time assay (Fig. 3A–E).

One of the non-GH proteins, CbHsp18, belongs to the small heat shock protein family. Small heat shock proteins can bind to exposed hydrophobic surfaces of partially unfolded proteins, by which they stabilize the target proteins in unfavorable chemical or physical environments [27]. Like other small heat shock proteins, CbHsp18 is a large oligomer at room temperature, and it dissociates into small oligomers exposing its hydrophobic surface when it is heated, e.g. at 70°C, which is reflected by the dynamic light scattering analysis of the particle radius of CbHsp18 in solution (Figure S1). Unlike CbHsp18, MkHistone1 belongs to histone proteins which specifically bind to DNA [28]. RNase A is a single-stranded RNA cleaving endonuclease, and hence the three proteins neither are related nor have glycoside hydrolase activities. Nevertheless, the extents of the stabilizing effects of CbHsp18 and MkHistone1 were similar, and although RNase A seemed to elicit a slightly weaker effect, the levels of enhancement for all three non-GH proteins were similar. In addition to increasing the thermostabilities of the three glycoside hydrolases tested, MkHistone1 and RNase A changed the initial product distribution patterns of cellopentaose hydrolysis by CbCdx1A. A circular dichroism analysis revealed that the gross structures of MkHistone1 and RNase A were slightly but statistically significantly changed, while that of CbHsp18 was not changed (Table S2). A possible explanation for the effects seen with the three proteins is that CbHsp18 protected the enzymes from unfolding via its chaperone activity, while MkHistone1 and RNase A created a sub-crowding environment, in which the thermophilic glycoside hydrolases were stabilized and hence leading to the increased end product yield. It is noteworthy that each of the non-GH proteins could stabilize more than one thermophilic glycoside hydrolases. In addition, while slight change of the gross structure of RNase A can be suggestive of some partial loss of its biological functions, the interpretation of the data for MkHistone1 may be different. MkHistone1 packages the genome of *Methanopyrus kandleri*, an archaeon that grows the best above 100°C. It is, therefore, likely that heating at a high temperature rather helps the protein to assume its proper fold. Thus the ability to stabilize the different GH proteins by the non-GH proteins may be different, with Hsp18 protein stabilizing through its chaperone activity, while the RNase A and the MkHistone1 stabilize through non-specific bindings, that could be hydrophobic interaction, electrostatic interaction and/or hydrogen bonding.

Other proteins or chemicals, such as bovine serum albumin (BSA) [29], corn stover hydrophobic proteins [30], Zea H [31], and polyethylene glycol (PEG) [32] have been applied in enhancement of plant cell wall hydrolysis. However, the mechanisms for the enhancing effects for these macromolecules were reported to derive from either a decrease in the non-productive adsorption of cellulases to the lignocellulose [29], [30], [32] or a change in the structure of the substrates [31]. Although the three non-GH proteins may have, to some extent, enhanced the activities of the glycoside hydrolases through the two previously described mechanisms, we demonstrate that all three proteins also increased the enzyme stability at high temperatures during end-over-end mixing. Moreover, the enzymes used in the previous studies referenced above were from mesophilic organisms. The three non-GH proteins investigated in the present study are small polypeptides derived from the three different lineages of life, i.e., Bacteria (CbHsp18), Archaea (MkHistone1), and Eukarya (RNase A), suggesting that a wide spectrum of proteins can be tested for this purpose. Moreover, we show that small polypeptides, which can be easily and cheaply produced in large quantities, can significantly enhance stability of thermostable enzymes and therefore yield of sugars for subsequent fermentation to value added products, such as biofuels. Note that although the pattern was not changed in the long period (24-h) of hydrolysis of Avicel by a combination of 2 cellulases of different function (CbCelA-TM1/CbCdx1A), the hydrolysis patterns of cellopentaose by CbCdx1A in presence of certain concentrations of MkHistone1 and RNase A in a short time reaction (9-min) were altered. This observation suggests that fine tuning experiments will be required to derive the best results for application of non-GH proteins as additives in the enzymatic hydrolysis of plant cell walls.

Two of the proteins (CpHsp18 and MkHistone1) introduced in the present report are from hyperthermophilic sources, and hence they can be easily expressed in *E. coli* cells and highly purified through a single purification (heating) step to remove the host proteins. Furthermore, as the effects of biomass hydrolysis enhancing chemicals such as PEG on down-stream fermentation by yeast and other ethanologenic organisms are not known, these non-glycoside hydrolase proteins may serve as alternatives in increasing the efficiency of the enzymatic hydrolysis of polysaccharides in biofuel production.

Enzyme cost represents one of the major setbacks in the conversion of cellulosic materials to biofuels. Hence factors that enhance hydrolytic activity and enzyme stability, which should facilitate enzyme recycling, will significantly impact the feasibility of the emerging biomass to biofuel industry.

## Materials and Methods

### Cloning of the Genes Encoding the *C. bescii* Proteins (Glycoside Hydrolases, a Small Heat Shock Protein) and the *M. kandleri* Histone1

The *Escherichia coli* strain XL10-Gold (Stratagene, Santa Clara, CA) was used for gene cloning, plasmid maintenance, and propagation throughout this study. The nucleotides encoding a truncation mutant (designated as TM1 in this study) of a cellulase CelA (GenBank accession number ACM60955), a putative β-1,4-glucosidase (GenBank accession number ACM59590, designated CbCdx1A in this study), a xylanase (GenBank accession number ACM59337, designated as CbXyn10A in this study), and a small heat shock protein of *C. bescii* (GenBank accession number ACM60607, designated CbHsp18 in this study) were amplified from the genomic DNA of *C. bescii* DSM 6725 by PCR using PrimeSTAR HS DNA Polymerase (Takara, Shiga, Japan) with gene specific primers (Table S1). The gene coding for a *Methanopyrus kandleri* histone (GenBank accession number AAM02890, designated as MkHistone1) was amplified from the *M. kandleri* genomic DNA by PCR also using PrimeSTAR HS DNA Polymerase and gene specific primers (Table S1). The PCR products were analyzed on a 1% agarose gel for their sizes. The bands with the expected sizes were cut out from the gel and the DNA was extracted from the gel using a QIAquick Gel Extraction kit (QIAGEN, Valencia, CA). The purified PCR products for *CbCelA-TM1*, *Cbcdx1A*, *Cbxyn10A*, and *Cbhsp18* were treated with the exonuclease activity of T4 DNA Polymerase and annealed to a pET-46 Ek/LIC plasmid according to the instruction of the manufacturer (Merck, Darmstadt, Germany). The annealing mixture was transformed into XL10-Gold competent cells by the heat-shock method and spread on Lysogeny broth (LB) agar plates supplemented with 100 µg/ml ampicillin. After overnight culture, single colonies were inoculated into 3-ml LB medium supplemented with 100 µg/ml ampicillin and cultured for 8 h. Plasmids were extracted using a QIAGEN Plasmid Mini kit and nucleotide sequenced to confirm the correct insertion of the target genes into the plasmid (W. M. Keck Center for Comparative and Functional Genomics, University of Illinois, Urbana). For MkHistone1, the extracted PCR product encoding the protein was treated with GoTaq DNA Polymerase (Promega, Madison, WI) in presence of 0.2 mM dNTP. The PCR product was then purified with a QIAquick PCR Purification Kit (QIAGEN, Valencia, CA) and cloned into a pGEM-T vector (Promega, Madison, WI). The insert was next restriction digested from the pGEM-T vector with NdeI and XhoI and cloned into a modified pET-28 plasmid (Merck, Darmstadt, Germany) (with the gene conferring kanamycin resistance replaced by that conferring ampicillin resistance) [33] already digested with the two restriction enzymes. The recombinant plasmid was sequenced to confirm the correctness of the inserted gene.

### Gene Expression and Protein Purification

The recombinant pET-46 Ek/LIC plasmids harboring the genes coding for CbCelA-TM1, CbCdx1A, CbXyn10A, and CbHsp18 and the pET-28 plasmid harboring the gene encoding MkHistone1 were transformed individually into *E. coli* BL21-CodonPlus (DE3)-RIPL competent cells (Stratagene, La Jolla, CA) and selected on LB agar plates supplemented with 100 µg/ml ampicillin and 50 µg/ml chloramphenicol. A single colony from each of the overnight culture was inoculated into 10 ml LB containing the same antibiotics and incubated at 37°C with shaking for 6 hr. This culture was transferred into a 1-liter LB medium containing the same concentration of both antibiotics and shaken at a speed of 250 rpm at 37°C until the optical density at 600 nm (OD_600_) reached 0.3. The temperature was decreased to 16°C, and isopropyl β-D-thiogalactopyranoside (IPTG) was added to the culture at a final concentration of 0.1 mM. The culture was continued at 16°C for 16 hr. The *E. coli* cells were harvested through centrifugation at a speed of 4651×g at 4°C for 15 min. The cell pellet was re-suspended in a binding buffer (50 mM Tris-HCl, 300 mM NaCl, pH 7.5) and then passed through an EmulsiFlex C-3 cell homogenizer (Avestin, Ottawa, Canada). The cell lysate was centrifuged at a speed of 12,857×g at 4°C for 20 min. The recombinant proteins in the supernatants were purified by immobilized metal ion affinity chromatography (IMAC) using a cobalt-charged resin (Talon Metal Affinity resin, Clontech, Mountain View, CA) according to the manufacturer’s instructions. The purified proteins were dialyzed against a storage buffer (50 mM Tris, 150 mM NaCl, pH 7.5) and stored until used.

### Preparation of Microwaved Miscanthus

Microwave has been shown to be an effective method in pretreatment of lignocellulose to enhance hydrolysis by glycoside hydrolases [34], [35]. Therefore, we applied this treatment to the bioenergy feedstock Miscanthus to facilitate hydrolysis. Five grams of Miscanthus were suspended in 50 ml 1% NaOH and mixed thoroughly by stirring. The slurry was treated using microwave for 10 min at a power level of 250 watts. The sample was centrifuged at 12,857×g at 4°C for 20 min. The pellet was collected and washed extensively with deionized water until the pH reached neutral. The pellet was then dried at 70°C and milled into a coarse powder with a mortar and pestle.

### Thermostability Assay

To determine if the non-GH proteins, i.e. CbHsp18, MkHistone1, and RNase A, have effects on the thermostability of CbCelA-TM1, CbCdx1A, and CbXyn10A during incubation with shaking, each of the *C. bescii* glycoside hydrolase at a final concentration of 1 µM was shaken end-over-end in a citrate buffer (50 mM sodium citrate, 150 mM NaCl, pH 6.0) in a total volume of 500 µl at 70°C. In another set of experiment, different concentrations (8 µM, 16 µM, and 32 µM) of CbHsp18, MkHistone1, or RNase A were added to each enzyme and subjected to the end-over-end incubation. For CbCelA-TM1, aliquots were taken at 3 hr, 6 hr, 12 hr, and 24 hr. For CbXyn10A, aliquots were taken at 1 hr, 2 hr, 4 hr, 6 hr, 8 hr, and 24 hr. For CbCdx1A, samples were taken after 24 hr.

The residual activities for each enzyme were measured at 70°C in the same citrate buffer. For CbCelA-TM1, the substrate used for determining the residual activities was sodium carboxymethyl cellulose (CMC), for CbCdx1A the substrate was *p*NP-β-D-glucopyranoside, and for CbXyn10A the substrate was birchwood xylan (BWX). The reaction time for CbCelA-TM1, CbCdx1A, and CbXyn10A were 20 min, 5 min, and 30 min, respectively. For CbCelA-TM1 and CbXyn10A, released reducing ends were measured using a *p*-hydroxybenzoic acid hydrazide (PAHBAH) method [36]. For CbCdx1A, release of *p*NP was measured with a Cary 300 UV-Visible Spectrophotometer (Agilent, Santa Clara, CA).

### Hydrolysis of Avicel Cellulose by CbCelA-TM1

To determine whether CbHsp18, MkHistone1, or RNase A has effects on hydrolysis of the model crystalline cellulose Avicel by the endoglucanase CbCelA-TM1, the substrate at a final concentration of 20 mg/ml was incubated with 1 µM of CbCelA-TM1 in a citrate buffer (50 mM sodium citrate, 150 mM NaCl, pH 6.0) at a total volume of 500 µl and shaken end-over-end at 70°C for 24 hr. Three different concentrations (8 µM, 16 µM, and 32 µM) for each non-GH protein (CbHsp18, MkHistone1, or RNase A) were then added to the reactions and the hydrolysis of Avicel repeated. At different time points (3 hr, 6 hr, 12 hr, and 24 hr), aliquots were taken out and the released reducing ends were measured using the PAHBAH method. The constituents of the reducing ends in the reaction mixture were further analyzed by high-performance anion exchange chromatography with pulsed amperometric detection (HPAEC-PAD) as described below.

### Hydrolysis of Birchwood Xylan and Miscanthus by CbXyn10A

To determine the effect of CbHsp18, MkHistone1, and RNase A on birchwood xylan (BWX, purchased from Sigma-Aldrich, St. Louis, MO) and Miscanthus hydrolysis by the xylanase CbXyn10A, the enzyme at a final concentration of 0.25 µM (for BWX) or 1 µM (for Miscanthus) was incubated with 20 mg/ml final concentration of BWX or Miscanthus in a citrate buffer (50 mM sodium citrate, 150 mM NaCl, pH 6.0) at a total volume of 500 µl. Three different concentrations (8 µM, 16 µM, or 32 µM) of CbHsp18, MkHistone1, and RNase A were added to the reaction mixture which was shaken end-over-end at 70°C. The reaction time was 1 min for BWX and 24 h for Miscanthus. At different time points (1 hr, 2 hr, 4 hr, 6 hr, 8 hr, and 24 hr), aliquots were taken from the Miscanthus hydrolysis reactions, and the released reducing ends were determined using the PAHBAH method. The constituents of the reducing ends in the reaction mixtures were also analyzed using HPAEC-PAD.

### Hydrolysis of Cellopentaose by CbCdx1A

To determine whether the three non-GH proteins have effects on cello-oligosaccharides hydrolysis by CbCdx1A, cellopentaose (G5) was used as a model substrate for CbCdx1A. Cellopentaose at a final concentration of 0.68 mM was incubated with 1 µM of CbCdx1A in the absence or the presence (8 µM, 16 µM, or 32 µM) of CbHsp18, MkHistone1, or RNase A in a total volume of 20 µl. The reaction was carried out for 9 min at 70°C on an Eppendorf Thermomixer (Hamburg, Germany) with a shaking speed of 300 rpm. After the incubation, the reaction products were immediately diluted 100-times in buffer A (100 mM NaOH) to terminate the reaction followed by analysis through the HPAEC-PAD method described below.

### Hydrolysis of Avicel by a Binary Enzyme Mixture Containing CbCelA-TM1 and CbCdx1A

Twenty mg/ml of Avicel was incubated with CbCelA-TM1 (1 µM final) and CbCdx1A (1 µM final) in the absence or the presence (16 µM, 32 µM, or 64 µM) of CbHsp18, MkHistone1, or RNase A in a citrate buffer (50 mM sodium citrate, 150 mM NaCl, pH 6.0) at a total volume of 500 µl. The reactions were carried out at 70°C for 24 hr with end-over-end shaking. The released reducing ends were measured using the PAHBAH method, and the constituents were analyzed using HPAEC-PAD.

### High-performance Anion Exchange Chromatography with Pulsed Amperometric Detection (HPAEC-PAD)

For HPAEC-PAD analysis, 100 µl of appropriately diluted hydrolysates were analyzed on a System Gold high-performance liquid chromatography (HPLC) instrument from Beckman Coulter (Fullerton, CA). The HPLC instrument was equipped with a CarboPac PA1 guard column (4 by 50 mm) and an analytical column (4 by 250 mm) from Dionex Corporation (Sunnyvale, CA) connected to a Coulochem III electrochemical detector from ESA Biosciences (Chelmsford, MA). The flow rate was 1 ml/min. Monomeric glucose and xylose, and the oligosaccharides cellobiose, xylobiose, and xylotriose were used as standards. The monosaccharides and oligosaccharides were resolved by using a mobile phase of 100 mM NaOH(Buffer A) with a linear gradient of Buffer B (100 mM NaOH, 1.1 M sodium acetate).

#### Analysis of subunit organization by dynamic light scattering (DLS)

The dynamic light scattering of CbHsp18 was conducted as described by Tarasov et al. [37] on a DynaPro Titan equipped with a Temperature-Controlled Microsampler (Wyatt Technology Corporations, Santa Barbara, CA) at a wavelength of 830 nm. CbHsp18 (0.2 mg/ml) in a phosphate buffer (10 mM sodium phosphate, pH 7.0) was filtered through a 0.22 µM membrane and then pre-incubated at temperature ranges from 25 to 85°C, at 5°C intervals, for 2 min. The dynamic light scattering signal was read at a laser power of 50%. The acquisition time was 15 s, with 10 times of acquisition. The intensity autocorrelation functions were analyzed by the software “Dynamics 6.9.2.11” (Wyatt Technology Corporations).

#### Circular dichroism spectroscopy

Far-UV CD spectroscopy was employed to analyze the secondary structures of the non-GH proteins CbHsp18, MkHistone1, and RNase A. The measurement was performed using a J-815 CD spectropolarimeter (Jasco, Japan) equipped with a constant temperature cell holder. The non-GH proteins (0.2 mg/ml in a pH 7.0 10 mM sodium phosphate buffer) were either kept static at 4°C or shaken end-over-end at 70°C for 24 h. Then the proteins were centrifuged at 25,000×*g* for 10 min to remove possible insoluble aggregates. The proteins were dispensed into a 1-mm quartz cuvette and kept at 25°C for 5 min before scanning. The measurements started with an initial wavelength of 260 nm and a final wavelength of 190 nm with a wavelength step of 0.1 nm. The analysis of the secondary structure of the proteins was conducted on the Dichroweb website (http://dichroweb.cryst.bbk.ac.uk/html/home.shtml) using an algorithm of CDSSTR with a reference Set 4 optimized for 190–240 nm [38], [39].

## Supporting Information

Figure S1 **Dynamic light scattering analysis of CbHsp18.** The dynamic light scattering of CbHsp18 was conducted on a DynaPro Titan equipped with a Temperature-Controlled Microsampler at a wavelength of 830 nm. CbHsp18 (0.2 mg/ml) in a phosphate buffer (10 mM sodium phosphate, pH 7.0) was filtered through a 0.22 µM membrane and then pre-incubated at a specific temperature (from 25 to 85°C, at 5°C intervals) for 2 min. The dynamic light scattering signal was read at a laser power of 50%. The acquisition time was 15 s, with 10 times of acquisition. The intensity autocorrelation functions were analyzed by the software “Dynamics 6.9.2.11”.
(TIF)

Table S1 **Primers used in this study.**
(DOC)

Table S2 **CD spectroscopy analysis of CbHsp18, MkHistone1, and RNase A.**
(DOC)
