# Supplementary figures and images for: Supplementing with Non-Glycoside Hydrolase Proteins Enhances Enzymatic Deconstruction of Plant Biomass
Source: PLoS One. 2012 Aug 27;7(8):e43828. doi: 10.1371/journal.pone.0043828 (PMC3428283; doi:10.1371/journal.pone.0043828)

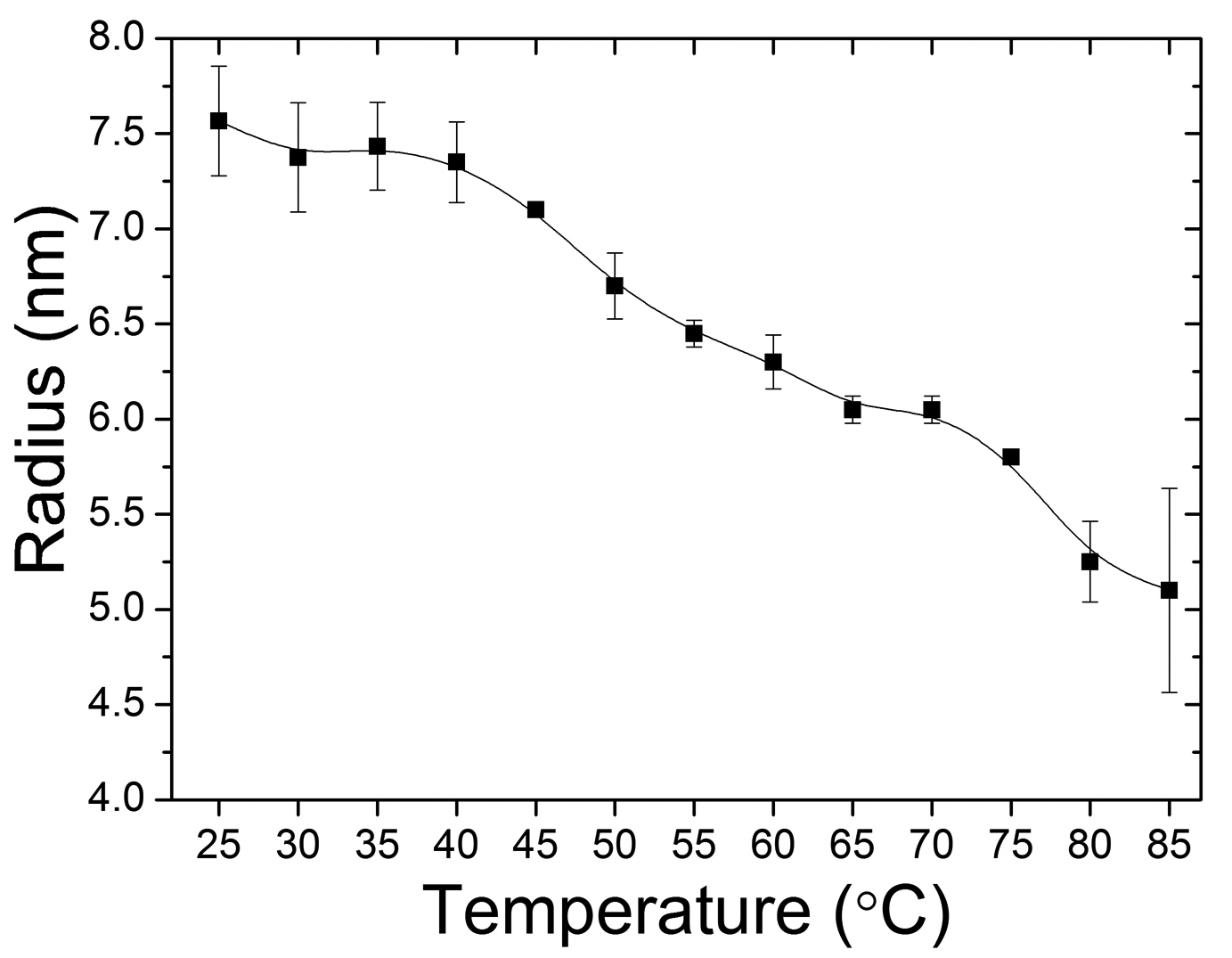

Supplement: Figure S1 — Dynamic light scattering analysis of CbHsp18. The dynamic light scattering of CbHsp18 was conducted on a DynaPro Titan equipped with a Temperature-Controlled Microsampler at a wavelength of 830 nm. CbHsp18 (0.2 mg/ml) in a phosphate buffer (10 mM sodium phosphate, pH 7.0) was filtered through a 0.22 µM membrane and then pre-incubated at a specific temperature (from 25 to 85°C, at 5°C intervals) for 2 min. The dynamic light scattering signal was read at a laser power of 50%. The acquisition time was 15 s, with 10 times of acquisition. The intensity autocorrelation functions were analyzed by the software “Dynamics 6.9.2.11”. (TIF) [file pone.0043828.s001.tif]
