# Supplementary material for: Supplementing with Non-Glycoside Hydrolase Proteins Enhances Enzymatic Deconstruction of Plant Biomass
Source: PLoS One. 2012 Aug 27;7(8):e43828. doi: 10.1371/journal.pone.0043828 (PMC3428283; doi:10.1371/journal.pone.0043828)
Supplement: Table S1 — Primers used in this study. (DOC) [file pone.0043828.s002.doc]

**Table S1. Primers used in this study**

| Protein | Primer direction | Primer sequence |
| --- | --- | --- |
| CbCelA-TM1 | Forward | 5'-GACGACGACAAGATGCAAGAGGTTAGGGCTGGTTCGTTTAAC-3' |
|  | Reverse | 5'-GAGGAGAAGCCCGGTTATACCTTTATCTGTCCACCTGCTAC-3' |
| CbCdx1A | Forward | 5'-GACGACGACAAGATGAGTTTACCAAAAGGATTTCTGTGGGGTGC-3' |
|  | Reverse | 5'-GAGGAGAAGCCCGGTTATGAGTTTTCCTTTATATACTGCTG-3' |
| CbXyn10A | Forward | 5'-GACGACGACAAGATGAACTTTGAAGGAAGAGAC-3' |
|  | Reverse | 5'-GAGGAGAAGCCCGGTTATTTTTTAGCCTTTAC-3' |
| CbHsp18 | Forward | 5'-GACGACGACAAGATGCTCAGAGACATAGTTCCATTTGGC -3' |
|  | Reverse | 5'-GAGGAGAAGCCCGGTTATTCTATATCAATTGTTCTTACATC -3' |
| MkHistone1 | Forward | 5'-CATATGGCCGTGGAGCTACCCAAGGCTGCCATCGAGAGGA-3' |
|  | Reverse | 5'-CTCGAGTTAGAGCTCTCCACCCTTGGGCATGGAGTACGTGA-3' |
