# Supplementary material for: Supplementing with Non-Glycoside Hydrolase Proteins Enhances Enzymatic Deconstruction of Plant Biomass
Source: PLoS One. 2012 Aug 27;7(8):e43828. doi: 10.1371/journal.pone.0043828 (PMC3428283; doi:10.1371/journal.pone.0043828)
Supplement: Table S2 — CD spectroscopy analysis of CbHsp18, MkHistone1, and RNase A. (DOC) [file pone.0043828.s003.doc]

**Table S2. CD spectroscopy analysis of CbHsp18, MkHistone1, and RNase A***

| **Protein** |  | **α-helix** | **β-sheet** | **Turn** | **Unordered** |
| --- | --- | --- | --- | --- | --- |
| CbHsp18 | 4°C | 11.8±1.5 | 35.3±1.0 | 23.3±0.5 | 30.3±1.3 |
|  | 70°C | 10.8±1.0 | 35.3±1.5 | 23.3±0.5 | 31.0±0.0 |
| MkHistone1 | 4°C | 52.5±1.3 | 13.0±1.6 | 11.5±1.7 | 22.3±1.0 |
|  | 70°C | 48.5±0.6*^p^*^<0.01^ | 13.8±1.0 | 13.0±0.8 | 24.5±1.0 *^p^*^=0.02^ |
| RNase A | 4°C | 14.3±0.5 | 29.3±0.5 | 24.3±0.5 | 31.8±1.3 |
|  | 70°C | 10.5±1.0 *^p^*^<0.01^ | 31.5±1.0 *^p^*^<0.01^ | 24.3±0.5 | 33.5±0.6 |

^*^: The values in the table are given as percentage. CbHsp18, MkHistone1, and RNase A were either kept static at 4°C (as a control) or shaken end-over-end at 70°C for 24 h. All values are expressed as mean±standard deviation from four independent measurements. The *p* values are given by comparison of the value at 70°C with that at 4°C using the *t*-test.
